# Honeybees fail to discriminate floral scents in a complex learning task after consuming a neonicotinoid pesticide

**DOI:** 10.1242/jeb.217174

**Published:** 2020-08-28

**Authors:** Julie A. Mustard, Annie Gott, Jennifer Scott, Nancy L. Chavarria, Geraldine A. Wright

**Affiliations:** 1 Department of Biology, University of Texas Rio Grande Valley, Brownsville, TX 78520, USA; 2 Institute of Neuroscience, Newcastle University, Newcastle upon Tyne NE2 4HH, UK; 3 Department of Zoology, University of Oxford, Oxford OX1 3SZ, UK

**Keywords:** Olfactory learning, Nicotinic acetylcholine receptor, Thiamethoxam, *Apis mellifera*, Odour discrimination, Cholinergic signalling

## Abstract

Neonicotinoids are pesticides used to protect crops but with known secondary influences at sublethal doses on bees. Honeybees use their sense of smell to identify the queen and nestmates, to signal danger and to distinguish flowers during foraging. Few behavioural studies to date have examined how neonicotinoid pesticides affect the ability of bees to distinguish odours. Here, we used a differential learning task to test how neonicotinoid exposure affects learning, memory and olfactory perception in foraging-age honeybees. Bees fed with thiamethoxam could not perform differential learning and could not distinguish odours during short- and long-term memory tests. Our data indicate that thiamethoxam directly impacts the cognitive processes involved in working memory required during differential olfactory learning. Using a combination of behavioural assays, we also identified that thiamethoxam has a direct impact on the olfactory perception of similar odours. Honeybees fed with other neonicotinoids (clothianidin, imidacloprid, dinotefuran) performed the differential learning task, but at a slower rate than the control. These bees could also distinguish the odours. Our data are the first to show that neonicotinoids have compound specific effects on the ability of bees to perform a complex olfactory learning task. Deficits in decision making caused by thiamethoxam exposure could mean that this is more harmful than other neonicotinoids, leading to inefficient foraging and a reduced ability to identify nestmates.

## Abstract (precis)

**Summary:** Honeybees exposed to the neonicotinoid pesticide thiamethoxam cannot perform a complex learning task. Honeybees exposed to other neonicotinoids such as imidacloprid and clothianidin, can learn this task, but at a slower rate.

## INTRODUCTION

Since the beginning of the 20th century, agriculture has increasingly relied on industrial chemicals that kill or repel insect pests, fungi, non-crop plants and plant pathogens. Thousands of tons of pesticides, herbicides and fungicides are applied to crops each year throughout the world (Carvalho, 2017). Pesticides are often applied to crops as a seed dressing; when the plant grows, the pesticides permeate the plant's tissues. Compounds that cannot be applied to seeds as systemic pesticides are instead applied through water sources or sprayed topically (Goulson, 2013; Bonmatin et al., 2015). While the main intention of the application of these substances to plants is to target pests, many of the compounds affect ‘non-target’ organisms such as pollinators like bees.

Bees encounter pesticides when they contact flower parts to drink nectar and collect floral pollen from flowering crops such as fruit, nut and seed crops. Consuming nectar and pollen exposes them to sublethal concentrations of these compounds that can accumulate in stored foods or in body tissues (Moffat et al., 2016; Osterman et al., 2019) and affect behaviours such as foraging (Henry et al., 2012; Muth and Leonard, 2019; Schneider et al., 2012; Yang et al., 2008), navigation (Fischer et al., 2014) and dance (Eiri and Nieh, 2016). Certain pesticides, such as neonicotinoids, are undetectable by bees when they are found in nectar, and so bees unwittingly consume these substances (Kessler et al., 2015). Several studies have now shown that populations of bee species such as bumblebees exposed to neonicotinoids in food are more likely to be reduced (Rundlöf et al., 2015).

An important cue that bees associate with the value of floral nectar is floral scent (Wright and Schiestl, 2009). A single pairing of scent with food is sufficient for bees to learn the odour is a signal of reward (Menzel and Müller, 1996). They selectively learn to forage on flowers of the same plant species to improve the efficiency of food collection (Menzel and Müller, 1996). Honeybees attend to subtle differences in scent such as the ratios of compounds in a floral perfume to identify flowering plant species with the best rewards (Wright et al., 2005, 2007, 2009). Bees can also learn to avoid odours associated with toxins in food (Wright et al., 2010).

The neonicotinoid class of pesticides activate the nicotinic acetylcholine receptor (nAChR), disrupting cholinergic signalling. Neurotransmission using acetylcholine takes place throughout the honeybee brain, including regions implicated in olfaction and learning and memory, such as the antennal lobes and mushroom bodies, as well as the suboesophageal ganglion, which receives gustatory information from sensory neurons on the proboscis (Kreissl and Bicker, 1989). Although neonicotinoids all act as agonists at nAChRs, they can affect receptor function in different ways. For example, imidacloprid (IMD) (Dupuis et al., 2011; Barbara et al., 2005) and dinotefuran (DNF) act as partial agonists (Tan et al., 2007). Clothianidin (CLO) is a full or super agonist, stimulating nAChRs to a greater degree than acetylcholine (Brown et al., 2006). Although thiamethoxam (TMX) may not directly bind to nAChRs, its actions may be due to the activity of its metabolic products, CLO and *N*-desmethyl-thiamethoxam, both of which act as agonists at nAChRs (Tan et al., 2007; Nauen et al., 2003). Because of their different affinities for the nAChR, it is likely that the effects of neonicotinoids cannot be extrapolated from studies of one compound, such as IMD, but that each compound may have different effects that could depend on the insect species studied.

Several studies have now confirmed that bees exposed to neonicotinoids for long periods at field-realistic concentrations (Dively and Kamel, 2012; Osterman et al., 2019; Rortais et al., 2005; Sanchez-Bayo and Goka, 2014) have difficulty learning to associate floral scent with food (Decourtye et al., 2004; Mengoni Goñalons and Farina, 2015; Piiroinen and Goulson, 2016; Tison et al., 2019; Williamson and Wright, 2013; Williamson et al., 2013; Zhang and Nieh, 2015). Most of this research has used simple, Pavlovian conditioning to study how neonicotinoid exposure affects the rate of olfactory learning to a food reward (but see Mengoni Goñalons and Farina, 2015; Zhang and Nieh, 2015; Stanley et al., 2015; Piiroinen et al., 2016). Few have compared bee performance in the same assays using several neonicotinoids. Here, we tested whether exposure to neonicotinoids in food affects the ability of honeybees to learn to discriminate among two scents signalling different rewards as they would during foraging. We found that bees exposed to TMX were completely unable to differentiate floral scents, whereas bees exposed to IMD, CLO or DNF were slower to learn the differences but still had the ability to differentiate odours.

## MATERIALS AND METHODS

### Honeybees

Honeybee colonies (*Apis mellifera* L.) from a Buckfast breeding population were obtained from the National Bee Unit, York, UK, and maintained at Newcastle University. Foragers were individually collected in glass vials at the colony entrance as they returned from foraging. Data for the differential learning experiments were collected from two colonies; data for the simple conditioning assay and for the gustatory test were collected from one colony. Honeybees (used in part of the gustatory assays only) at the University of Texas Rio Grande Valley were purchased from ETzzz Bzzz (College Station, TX, USA) and maintained on the University of Texas Rio Grande Valley campus in Brownsville, TX, USA.

### Pesticide treatment

Neonicotinoid pesticides (obtained from Sigma-Aldrich) were dissolved directly in 1 mol l^−1^ sucrose syrup for oral administration to bees. The neonicotinoids were serially diluted from a 10 µmol l^−1^ stock solution to create the final concentrations used in these experiments (see Table 1). Forager bees were collected from the hive entrance in small plastic vials. The bees were cold anaesthetized then transferred in groups of 20 bees to rearing cages (16.5 cm×11 cm×6.5 cm) as described in Williamson et al. (2014). At least five cages per treatment were used. Food laced with the pesticide treatment was delivered using 2 ml microcentrifuge tubes with four evenly spaced 2 mm holes. The feeders were inserted through holes cut in the sides of the boxes. The bees subjected to differential conditioning were allowed to feed *ad libitum* on 10 nmol l^−1^ concentration of each pesticide solution for 24 h (see Table 1 for doses per bee). This concentration was chosen based on the range reported from the nectar of seed-treated and sprayed plants (see ‘Extended Data Table 1’ in Kessler et al., 2015). The next day, each bee was anaesthetized on ice and placed in a harness as described in Wright et al. (2007). Each bee was fed with 20 µl of the pesticide it experienced prior to conditioning, and left on the bench for 16–24 h prior to conditioning experiments. After conditioning, each bee was fed 20 µl of the same pesticide treatment solution as before, and left for 24 h prior to the long-term memory test. The total average doses each bee received for each pesticide over the course of the experiment was between 1.9 and 2.7 ng per bee, depending on the pesticide (Table 1). [Note, the data for TMX were lost, and so an average volume based on all the other pesticides was calculated for this treatment. Our previous work using the same methods showed that bees do not eat significantly different volumes of 10 nmol l^−1^ concentrations of these pesticides in 1 mol l^−1^ sucrose in a no-choice setting (see Williamson et al., 2014; Table 1)]. During *ad libitum* feeding, the total amount of food consumed by each bee was not significantly different among the treatments, including the control (one-way ANOVA, *F*_3,85_=0.182, *P*=0.908). The bees subjected to simple conditioning or the gustatory test were fed with 20 µl of 10 nmol l^−1^ pesticide solution 24 h prior to use.

**Table 1.**
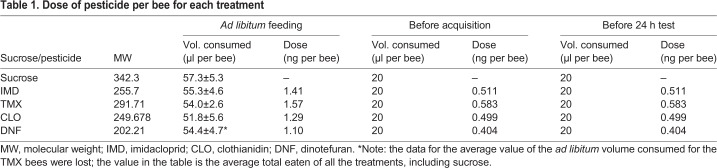
**Dose of pesticide per bee for each treatment**

### Conditioning

Differential conditioning was carried out as in Wright et al. (2008), except that 10 mmol l^−1^ quinine was used as a negative stimulus rather than salt solution. Bees were conditioned to learn to associate distinct olfactory conditioned stimuli (CS) with a positive reinforce or a punishment (unconditioned stimulus, US): the odour 1-hexanol (CS+) was paired with a positive reinforcer of 0.4 µl of 1 mol l^−1^ sucrose, whereas the odour 1-decanol (CS−) was paired with the punishment of 0.4 µl of 10 mmol l^−1^ quinine (a saturated solution). The sucrose or quinine solution was delivered using a Gilmont syringe (Cole-Parmer). The odour stimulus was 3 µl of odour (Sigma-Aldrich) placed on a strip of filter paper in a glass tube. Air was passed across the filter paper and delivered to the antennae of the bee for 4 s as previously described (Wright et al., 2007). The order of odour presentation was pseudorandomized, such that each trial with the CS+ and CS− could not be easily predicted by the subject (i.e. CS+, CS−, CS−, CS+, CS−, CS+, CS+, CS−, CS+, CS−, CS−, CS+). Each CS was presented for 6 trials (total of 12 trials) and the inter-trial interval was 5 min. For simple conditioning, bees were conditioned to learn to associate an olfactory stimulus (CS) with 0.4 µl of 1 mol l^−1^ sucrose US. Bees were conditioned over 6 trials with a 5 min inter-trial interval.

Bees trained in the differential learning task were tested with the CS+ (1-hexanol) and the CS− (1-decanol) and three other odours that form a perceptual gradient between 1-hexanol and 1-decanol (Wright et al., 2008, 2009). Memory tests were performed 10 min and 24 h after conditioning. The memory test was an unreinforced test with a series of the following odours: 1-hexanol (CS+), 1-heptanol, 1-octanol, 1-nonanol and 1-decanol (CS−). The order of presentation of the test odours was randomized across subjects. Each treatment group was randomized across the course of the study; on any given day, at least three treatment groups were trained and tested. Following simple conditioning, individual bees were only tested at 10 min after conditioning as described above.

### Gustatory test

Foragers were captured at the colony entrance, harnessed as described above, and fed to satiation with 1 mol l^−1^ sucrose. The next day, bees were fed with 20 µl of 10 nmol l^−1^ pesticide. Twenty-four hours later, bees were tested for their ability to sense 1 mol l^−1^ sucrose and 1 mol l^−1^ sucrose+10 mmol l^−1^ quinine. The antennae of each bee were touched with 1 mol l^−1^ sucrose to elicit the proboscis extension reflex (PER). The test solution was applied to the mouthparts to assess whether each bee would drink the droplet. Drinking was scored as 0 when it was refused and 1 when it was consumed, as described in Wright et al. (2010). In addition, the bees from the simple conditioning task (see ‘Conditioning’, above) were also tested for their responses to 1 mol l^−1^ sucrose and 1 mol l^−1^ sucrose+10 mmol l^−1^ quinine and to 10 mmol l^−1^ quinine immediately following their 10 min memory test.

### Data analysis

Data were recorded as a binary variable, where proboscis extension in response to CS presentation was scored as 1, and failure to extend the proboscis was scored as 0. Data for both the learning tasks and the memory tests were analysed using SPSS v23 using generalized estimating equations (GEE) for repeated measures as a binary logistic regression analysis (lreg) or a linear dependent variable (ldv). All bees failed to respond on the first trial of the CS+. To test how treatment affected the rate of acquisition of learning, data from the first trial with the CS+ and the CS− were excluded from the analysis to facilitate model fitting. *Post hoc* comparisons were made using Šidák’s test for comparisons against a control or as least-squares differences (lsd) pairwise comparisons. Analysis of the *ad libitum* consumption data was performed using one-way ANOVA. Data for the gustatory assay were tested using a generalized linear model (GLM). Data can be accessed at from the figshare digital repository (https://doi.org/10.6084/m9.figshare.8984225.v1).

## RESULTS

### Differential olfactory learning is impaired by pre-exposure to neonicotinoids

**Fig. 1.**
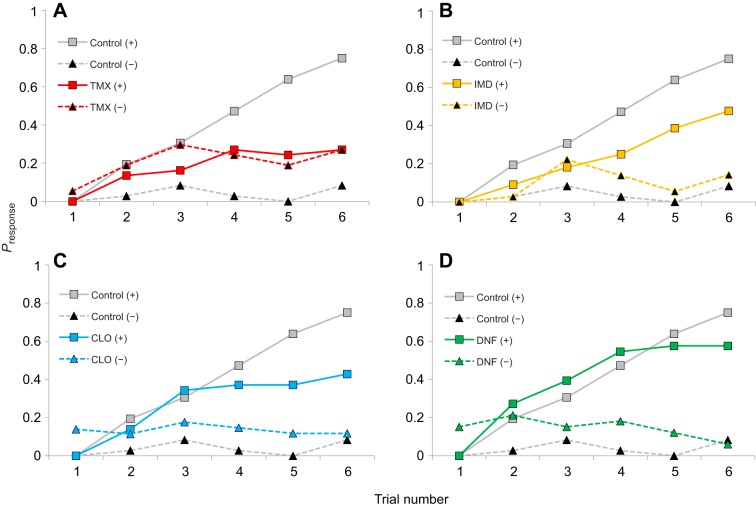
**Honeybees fed with neonicotinoids have difficulty performing a differential learning task.** (A) Thiamethoxam (TMX)-fed bees cannot distinguish the CS+ odour from the CS− odour after 12 trials of conditioning. Bees fed with imidacloprid (IMD; B), clothianidin (CLO; C) or dinotefuran (DNF; D) eventually learn the task, but they are slower than the bees in the control treatment (grey lines in all panels). The conditioned stimulus (CS) was reinforced with 1 mol l^−1^ sucrose (+) or 10 mmol l^−1^ quinine (−). *P*_response_ indicates the probability of proboscis extension reflex during odour stimulation prior to the delivery of the reinforcer. *N*_control_=36, *N*_IMD_=44, *N*_TMX_=37, *N*_CLO_=36, *N*_DNF_=33.

We used a well-established method to study how bees learn to distinguish odours by their respective outcomes. As expected, bees in the control treatment fed sucrose were readily able to learn that 1-hexanol (CS+) was associated with a sucrose reward whereas 1-decanol (CS−) was associated with quinine punishment (Fig. 1). Their responses to the CS+ and the CS− were significantly different by the second conditioning trial (Table 2; lsd, *P*=0.007).

**Table 2.**
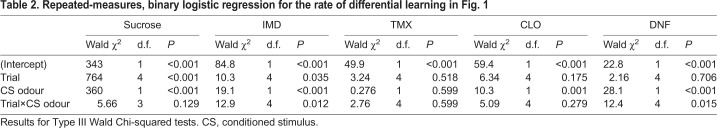
**Repeated-measures, binary logistic regression for the rate of differential learning in Fig. 1**

Honeybees fed for 48 h with a field-relevant concentration of a neonicotinoid pesticide, however, had difficulty performing the olfactory, differential learning task (Fig. 1). The magnitude of this effect depended on the specific neonicotinoid fed to the bees (Table S1; GEE, treatment×odour, χ_4_^2^=55.2, *P*<0.001). Bees fed with TMX could not differentiate the CS+ from the CS− during conditioning on any trial (Fig. 1A, Table 2). Bees fed with IMD or CLO were able to distinguish the CS+ and the CS−, but it took them longer to do so than the control bees (Fig. 1B,C). The IMD-fed bees were able to distinguish the CS+ and the CS− odours by the fifth trial (Table 2; lsd, *P*<0.001), whereas the CLO-fed bees were able to make this distinction on the fourth trial (Table 2; lsd, *P*=0.025). Bees fed with DNF (Fig. 1D) distinguished the CS+ from the CS− on the third trial (Table 2; lsd, *P*=0.033).

### TMX-exposed bees cannot distinguish odours

**Fig. 2.**
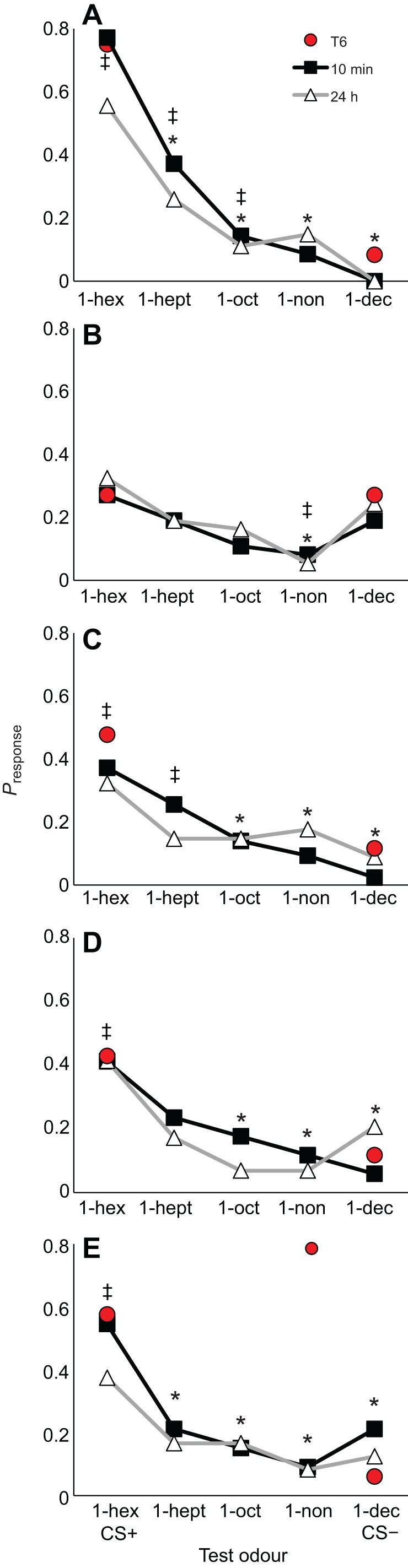
**Neonicotinoid pesticide exposure reduces olfactory acuity of adult worker honeybees.** For comparison, the response level at trial 6 (T6) for the CS+ and CS− is shown in each panel. (A) Control honeybees. (B) TMX-fed honeybees. Except for the novel odour, 1-nonanol, the TMX-fed honeybees responded to all the test odours at rates that were not significantly different from those for the CS+ and CS−. (C,D) The IMD-fed bees (C) and the CLO-fed bees (D) responded to the test odours in a manner similar to the control bees, but the slope of the gradient was much shallower and fewer of the responses to the novel odours were significantly different from those to the CS+ and CS−. (E) Bees fed with DNF responded least to the novel odours and the CS−; the responses to the novel odours were not significantly different from those to the CS−. *P<0.05 compared with CS+; ^‡^*P*<0.05 compared with the CS−. *N*_control_=36, *N*_IMD_=44, *N*_TMX_=37, *N*_CLO_=36, *N*_DNF_=33. 1-hex, 1-hexanol; 1-hept, 1-heptanol; 1-non, 1-nonanol; 1-oct, 1-octanol; 1-dec, 1-decanol.

Bees trained in the differential learning task were tested with a gradient of odours to test whether exposure to neonicotinoids affected olfactory perception and generalization (Fig. 2). Bees in the control treatment responded to the test odours in a way that was predicted by the perceptual similarity of the test odour to the CS+ and the CS− (Fig. 2A). The highest probability of eliciting a PER was towards the CS+; none of the control bees responded to the CS−. The response to the novel odours (1-heptanol, 1-octanol and 1-nonanol) was graded as predicted by previous studies (Wright et al., 2008).

Bees exposed to neonicotinoids in food prior to conditioning and testing had difficulty distinguishing odour stimuli (Fig. 2B–E). The responses of the bees were not significantly different at 10 min and 24 h (GEE, neonicotinoid×odour×time: χ_15_^2^=9.48, *P*=0.851; Table 3), but their responses to the test odours depended on the neonicotinoid treatment (GEE, neonicotinoid×odour: χ_15_^2^=3055, *P*<0.001; Table 3). The test gradient of the TMX-fed bees was flat compared with that of the control bees, indicating that the bees could not distinguish the novel odours from the CS+ and the CS− (Fig. 2A). The response to the CS+ and the response to the CS− were not significantly different for these bees. Bees fed with IMD and CLO could distinguish the novel odours from the CS+ (Fig. 2B,C), but the responses to the novel odours compared with that to the CS− were not significantly different (except for IMD-fed bees tested with 1-heptanol). This gradient was similar for the DNF-fed bees; these bees could clearly distinguish the CS+ from the novel odours, but the responses to the novel odours and the CS− were not significantly different (Fig. 2E).

**Table 3.**
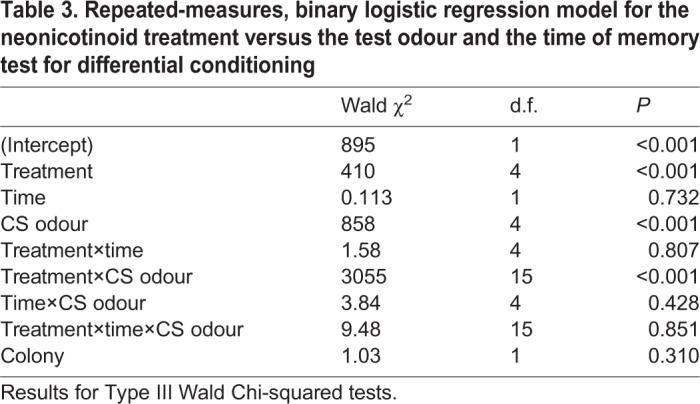
**Repeated-measures, binary logistic regression model for the neonicotinoid treatment versus the test odour and the time of memory test for differential conditioning**

To determine whether the impairments in the rate of differential learning were due to a change in the bees' gustatory perception, we fed bees with each of the pesticides (as before) and tested whether they would drink the quinine solution. Bees had no difficulty in distinguishing sucrose solution from sucrose laced with quinine, regardless of the pesticide tested (GEE, neonicotinoid×solution: χ_4_^2^=4.23, *P*=0.325; Table S2; *N*_control_=30, *N*_IMD_=26, *N*_TMX_=27, *N*_CLO_=27, *N*_DNF_=25).

### TMX-exposed bees learn but have impaired olfactory processing

**Fig. 3.**
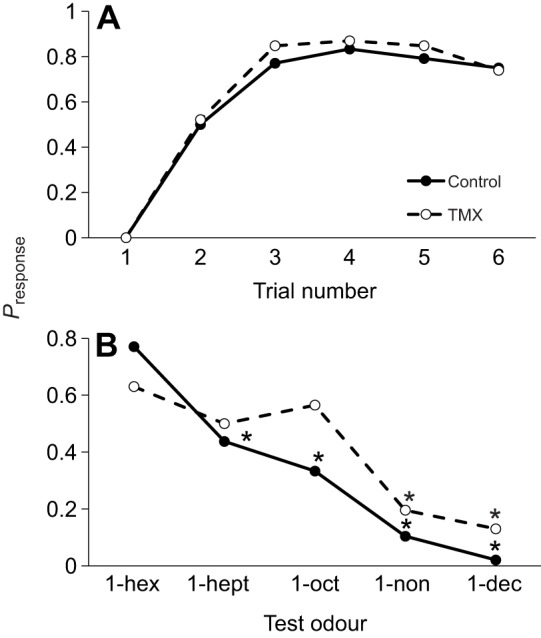
**TMX impairs olfaction not learning performance.** (A) TMX-fed honeybees did not exhibit impaired performance in a simple learning task. (B) Bees fed with TMX 24 h prior to conditioning and testing were unable to detect the difference in 3 out of 5 test odours during a short-term memory test, 10 min after conditioning. *P<0.05 compared with CS+. *N*_control_=64, *N*_TMX_=71.

The impairment of performance of the TMX-fed bees could be the result of impairment of associative learning, compromised ability to taste or impairment of olfactory sensation. To identify how TMX affected learning, we also fed a separate group of bees with 10 nmol l^−1^ TMX and trained them to learn to associate one odour with a food reward for six trials in a simple olfactory conditioning task. The TMX-fed bees did not differ significantly from the control bees in their performance during the six conditioning trials (Fig. 3A; GEE, treatment×trial: χ_4_^2^=0.816, *P*=0.936; Table S3; note: there was no difference in non-responders between the two treatments, Mann–Whitney, *Z*=−1.28, *P*=0.199). This indicates that TMX did not interrupt the ability to associate an odour with sucrose solution.

To identify whether TMX disrupted olfactory perception, each bee trained in Fig. 3A was tested 10 min after conditioning with the same suite of odours from Fig. 2. Bees fed with TMX were more likely to generalize to the other test odours (Fig. 3B; GEE, treatment×trial: χ_4_^2^=13.6, *P*=0.009; Table S4). Specifically, they exhibited responses to 1-heptanol and 1-octanol that were not significantly different from those to the CS (1-hexanol) (Šidák's test, *P*>0.05). The TMX-fed bees' responses to 1-nonanol and 1-decanol were significantly lower than their response to the CS (Šidák's test, *P*<0.001). This is in contrast to the control bees, which could easily distinguish each odour (Šidák's test, all *P*<0.001). We also verified the results of the gustatory assay by testing each subject for its response to the sucrose solution and to the quinine solution; TMX exposure did not have a significant effect on the response to sucrose or quinine (lreg, treatment×solution: χ_2_^2^=0.00, *P*=1.0).

## DISCUSSION

These data are the first to show that the neonicotinoid TMX completely ablates the ability of bees to learn to associate odours with different outcomes. These bees generalized among odours during the 10 min and 24 h memory tests as if they could not distinguish the CS+ from the CS−, indicating that they could not learn the different odour–outcome associations or could not distinguish the odours. A further experiment revealed that the ability of TMX-fed bees to perform a simple learning task was unaffected, but olfactory perception of similar odours was impaired. The combination of these data indicates that field-relevant doses of TMX affect both cognition and olfactory processing in honeybees. As expected from previous studies, bees fed with IMD or CLO were slower to differentiate the two odours, but they could still perform the learning task and were able to generalize to novel odours in a way that reflected what they had learned during conditioning. Bees fed with DNF did not have difficulty performing differential learning and generalized to novel odours in a way predicted by the control during the memory test.

If consumption of the pesticides produced changes in the gustatory perception of either unconditioned stimulus used in our experiments (i.e. sugar or quinine), it would be more difficult for bees to discriminate between the appetitive and aversive stimulus. Previous studies have found that bees fed with TMX, IMD or CLO have a reduced probability of eliciting the PER when the antennae are touched with low concentrations of sucrose (e.g. 3% w/v) but not with high concentrations (e.g. 30% w/v; Aliouane et al., 2009; Demares et al., 2018; Eiri and Nieh, 2016; Mengoni Goñalons and Farina, 2015, 2018). Based on these published studies, we expected that perception of the unconditioned stimuli in our experiments would not be affected by neonicotinoid exposure, as we used very concentrated sucrose as one of our unconditioned stimuli (34% w/v sucrose). No previous research has examined how neonicotinoids affect a bee's detection of bitter compounds like quinine. Our data clearly show that the gustatory perception on the mouthparts towards the unconditioned stimuli in our learning assays was not affected by the pesticide treatment. Thus, the deficit in the ability to perform differential learning is not a result of gustatory perception but is probably due to the impact of the neonicotinoids on nAChRs in the central nervous system.

Assigning different meanings to two stimuli of the same modality during a learning task has been described as a form of working memory in bees (Zhang et al., 2005). Many studies have shown impairments of Pavlovian olfactory conditioning in neonicotinoid exposed bees, but very few have looked at more complex forms of learning. None have compared more than one neonicotinoid in the same study in bees exposed to neonicotinoids over several days. In our experiments, the bees that were exposed to TMX had difficulty performing a complex learning task that required assigning meaning to two different odours. The TMX-exposed bees did not have problems learning during simple conditioning (as observed previously by Aliouane et al., 2009). Our data, therefore, indicate that the impact of TMX is specifically on circuits involved in the decision making required to recognize and respond appropriately to two different stimuli during a learning task. In *Drosophila*, this process requires the engagement of specific subsets of dopamine neurons that are arranged in a feed-forward inhibitory network which encodes the relative valence of learned stimuli (Cognigni et al., 2018). The influence on working memory must occur through the interactions of TMX with nAChRs found in mushroom bodies, as the neurons in this location gate memory and decision making in the insect brain (Barnstedt et al., 2016). Relatively little is known about the cell-specific expression of nAChR subunits in the bee brain. Although all the nAChR subunits are expressed its brain, only the subunits of the Kenyon cells have been reported, and they are different to those in the antennal lobes (Dupuis et al., 2011). Interestingly, an acute dose of TMX fed to bumblebees impairs their spatial working memory (Samuelson et al., 2016). The impact of TMX on cognitive processes in bees, therefore, may not require exposure over an extended period.

Bees exposed to IMD or CLO required more training trials to solve the discrimination learning task, but eventually could perform it. Unlike those exposed to TMX, their olfactory processing of odour identity during the memory tests was unaffected. Similar results for the impact of IMD on differential learning in bees have been obtained in other studies. Bumblebees fed an acute dose of IMD prior to a task had difficulty learning to distinguish artificial flowers with different scents (Muth and Leonard, 2019). Young worker honeybees between 5 and 14 days old exposed to IMD over longer periods (5–14 days) had slower rates of learning in both simple and differential learning tasks (Mengoni Goñalons and Farina, 2018). Others have found that IMD at a similar dose reduces the rate of acquisition during aversive learning using cues mimicking a predatory attack (Tan et al., 2014; Zhang and Nieh, 2015). Thus, IMD in general reduces the rate of learning in a population but does not completely impair the performance of bees. In addition, it is important to note that other studies of the impact of IMD on simple conditioning failed to find an effect of CLO or IMD at the doses we used during simple conditioning (Williamson et al., 2013; Tison et al., 2019). Because honeybees are excellent olfactory learners, it may require more demanding tasks, such as differential learning, to reveal deficits due to pesticide exposure at low, field-relevant doses. Learning and memory are essential for many honeybee behaviours, such as navigation, learning about flowers that provide food, and recruitment of other foragers via dancing (Menzel and Müller, 1996); even subtle disruption of learning or decision making may have a significant long-term impact on honeybee colonies.

Our simple conditioning experiment revealed that the effect of TMX on the test responses was in part due to a change in olfactory processing. This was shown by the change in the generalization gradient towards all the test stimuli. In the case of honeybees that had experienced simple conditioning, four out of five of the test odours they experienced were novel. Control bees exhibited a clear gradient in their responses to the test odours, where most bees responded to the conditioned odour, and fewer responded to the other odours. In these honeybees, the rate of response to the novel odours was proportional to perceptual differences in the odour stimuli relative to the rewarded odour, as expected (Daly et al., 2001; Wright et al., 2008). What is striking about the TMX-exposed honeybees in these experiments is that they responded to three out of five of the test odours as if all predicted the reward. In this case, because no other training was employed prior to the test, it is reasonable to assume that the increase in generalization of the conditioned response towards these novel but similar smelling odours is due to a change in the way the odours are perceived as a result of TMX exposure.

Cholinergic signalling is used in the entire olfactory circuit in the insect central nervous system so it is not surprising that olfactory function could be disrupted or affected by neonicotinoid pesticides (Barnstedt et al., 2016). Previous studies of the antennal lobe network illustrated that disruption of inhibition resulted in an impaired ability to sense differences in perceptually similar odours (Stopfer et al., 1997). When the temporal pattern of output from the projection neurons is impaired by the injection of a GABA antagonist into the antennal lobe, bees fail to distinguish 1-hexanol from 1-heptanol and 1-octanol (Stopfer et al., 1997). Using the same odours and simple conditioning assay, we found that bees exposed to TMX also failed to differentiate these odours. This implies that affecting cholinergic signalling in the antennal lobe also results in a change in the output from the projection neurons that encodes odour identity. Long-term exposure to a neonicotinoid could cause nAChR desensitization (Dupuis et al., 2011), altering the balance of excitation and inhibition that is necessary to encode olfactory information (Laurent, 2002). A calcium imaging study of the honeybee antennal lobes revealed that IMD reduced activation of the projection neurons but no changes in firing patterns could be resolved using this method (Andrione et al., 2016).

The differences we observed for the impact of neonicotinoids on olfactory learning and memory in bees show that these compounds have different pharmacological affinities for nAChRs. The nAChR channel is formed from the association of five subunits, and channels may contain the same (homomeric) or a mix (heteromeric) of different subunits. The combinations of different subunits produce nAChR variants with distinct pharmacological profiles. The honeybee genome contains 11 genes for putative nAChR subunits, the transcripts of which are further diversified by alternative splicing and RNA editing (Jones et al., 2006). The variation in expression of distinct subunits in different tissues and developmental stages (Dupuis et al., 2011; Thany et al., 2005) may lead to distinct properties for nAChRs involved in different processes. For example, antennal lobes contain at least two distinct nAChR subtypes (Barbara et al., 2008). Furthermore, antennal lobes and Kenyon cells express different sets of the nAChR subunits, and the nAChRs exhibit differences in properties such as desensitization (Dupuis et al., 2011). Additionally, calcium imaging of cultured bumble bee Kenyon cells revealed that, while all cells responded to acetylcholine, specific subsets of cells only responded to IMD or CLO. Both the mushroom bodies and the antennal lobes are involved in olfactory processing, and the variation in affinity of the nAChRs expressed in each tissue for different compounds may underlie the distinct effects observed on learning and olfaction in our experiments.

TMX exhibits very low binding to nAChR and is not effective at producing excitatory currents as a result of activation of nAChRs (Nauen et al., 2003; Tan et al., 2007). It has been suggested that it is the conversion of TMX into its metabolite CLO that is responsible for the efficacy of TMX (Nauen et al., 2003; Coulon et al., 2018; Moffat et al., 2016). However, this is hard to explain from our experiments and others, where CLO and TMX do not produce the same behavioural effects in bees (e.g. Williamson et al., 2014; Kessler et al., 2015; Moffat et al., 2016). Why then does TMX have a distinct effect from that of CLO? One possibility is that even low levels of metabolites of TMX other than CLO (e.g. *N*-desmethyl-thiamethoxam) may contribute to the distinct activity of TMX. Another possibility is that TMX is active at a specific form of bee nAChRs that are as yet unidentified. In the cockroach, TMX is preferentially active at the desensitizing form of the nAChR over the non-desensitizing form (Nauen et al., 2003). Recording from honeybee Kenyon cells in a whole-brain preparation, Palmer et al. (2013) showed that IMD and CLO lead to an initial increase in nAChR activity followed by desensitization of nAChRs and a reduction in excitatory current evoked by acetylcholine. By binding preferentially to the desensitizing form of nAChR, TMX could impact nAChR signalling, even though it has an overall low affinity for nAChR receptors.

The four compounds studied here produced distinct effects on olfaction and a complex task, differential learning, emphasizing that each neonicotinoid needs to be evaluated independently for its effects on honeybee behaviour. Although it is possible that the effects of TMX were a result of its slightly larger dose in our study (see Table 1), it is more likely that the effects are specific to this compound. Decision making is essential for the survival of all animals, insects included, and is especially important to foraging honeybees. Olfaction is essential for efficient foraging because it allows honeybees to predict which flowers provide high-quality food (Menzel and Müller, 1996). Bees are also able to learn to associate odours with toxic substances, even if they are unable to taste the toxin (Wright et al., 2010). The ability to distinguish subtle differences in scent is also essential for colony functioning, as bees identify nestmates by scent (Breed et al., 1988). Therefore, compounds such as TMX that interfere with decision making and olfactory processing would be expected to significantly impact the ability of honeybee colonies to accrue resources, avoid toxins and form a cohesive social group, negatively affecting their long-term survival.

## Supplementary Material

Supplementary information
