# Supplementary material for: Honeybees fail to discriminate floral scents in a complex learning task after consuming a neonicotinoid pesticide
Source: J Exp Biol. 2020 Aug 28;223(5):jeb217174. doi: 10.1242/jeb.217174 (PMC7075050; doi:10.1242/jeb.217174)
Supplement: Supplementary information [file jexbio-223-217174-s1.pdf]

Table S1. Repeated-measures, generalized estimating equations model for the neonicotinoid treatment vs. the odour-reinforcer pairing for the acquisition data in Figure 1.

	Type III		
	Wald χ^2	df	P-value
(Intercept)	361	1	< 0.001
CS odour	92.5	1	< 0.001
Treatment	10.5	4	0.033
CS odour x Treatment	55.2	4	< 0.001
Colony	0.187	1	0.665

Table S2. Repeated-measures, binary logistic regression model for the test of the bees tested with 1 M sucrose solution vs. 1 M sucrose solution laced with 10 mM quinine after exposure to a neonicotinoid pesticide (IMD, TMX, CLO, DNF) or the control.

	Type III		
	Wald χ^2	df	P-value
(Intercept)	582	1	< 0.001
Treatment	0.751	4	0.945
Test odour	222	4	< 0.001
Treatment x Test odour	4.24	4	0.375

Table S3. Repeated-measures, binary logistic regression model for the simple conditioning of TMX vs a control with no pesticide.

	Type III		
	Wald χ^2	df	P-value
(Intercept)	1070	1	< 0.001
Treatment	0.374	1	0.541
Trial	35.8	4	< 0.001
Treatment x Trial	0.816	4	0.936

Table S4. Repeated-measures, binary logistic regression model for the test of the bees subjected to simple conditioning after exposure to TMX or the control.

	Type III		
	Wald χ^2	df	P-value
(Intercept)	55.5	1	< 0.001
Treatment	1.65	1	0.199
Test odour	67.6	4	< 0.001
Treatment x Test odour	13.6	4	0.009

Journal of Experimental Biology • Supplementary information
